# Colorectal Hyperplasia and Dysplasia Due to Human Carcinoembryonic Antigen (CEA) Family Member Expression in Transgenic Mice

**DOI:** 10.1371/journal.pone.0001353

**Published:** 2007-12-26

**Authors:** Carlos H. F. Chan, Pilar Camacho-Leal, Clifford P. Stanners

**Affiliations:** McGill Cancer Centre, Department of Biochemistry, McGill University, Montréal, Québec, Canada; University of Birmingham, United Kingdom

## Abstract

CEA and CEACAM6 are immunoglobulin family intercellular adhesion molecules that are up-regulated without structural mutations in approximately 70% of human cancers. Results in *in vitro* systems showing tumorigenic effects for these molecules suggest that this correlation could indicate an instrumental role in tumorigenesis. To test whether this applies *in vivo*, transgenic mice harboring 187 kb of the human genome containing four CEA family member genes including the CEA and CEACAM6 genes were created and their copy numbers increased by mating until colonocyte expression levels reached levels seen in human colorectal carcinomas. The colonocyte surface level of integrin α5 and the activation of AKT increased progressively with the expression levels of CEA/CEACAM6. Colonic crypts showed a progressive increase in colonocyte proliferation, an increase in crypt fission, and a strong inhibition of both differentiation and anoikis/apoptosis. All transgenic mice showed massively enlarged colons comprising a continuous mosaic of severe hyperplasia, dysplasia and serrated adenomatous morphology. These results suggest that up-regulated non-mutated adhesion molecules could have a significant instrumental role in human cancer.

## Introduction

The human CEA family of cell surface intercellular adhesion molecules, a sub-family of the Immunoglobulin Superfamily, consists of both glyco-phosphatidylinositol (GPI) and transmembrane (TM) anchored members [1]. GPI-anchored members, CEA and CEACAM6, show up-regulation in 50–70% of all human cancers, including colon, breast and lung cancers [1]–[4]. In fact, CEA represents a major tumor marker used widely in the management of colorectal cancer [5]–[7]. The TM-anchored member, CEACAM1, on the other hand, usually shows down-regulation in cancers [1]. The question arises as to whether or not the positive correlation between CEA/CEACAM6 levels and tumors points to an instrumental role for these molecules in cancer. This question has significance for the clinical interpretation of CEA and CEACAM6 levels in tumors and for the development of CEA-targeted therapies. In spite of the fact that CEA and CEACAM6 are normally up-regulated as differentiation proceeds during the movement of colonocytes up colonic crypts [4], [8], experiments with various model systems *in vitro*, including cultured human colorectal carcinoma colonocytes, have shown that if CEA and/or CEACAM6 are inappropriately up-regulated in cells with division potential *prior* to differentiation, differentiation is blocked [9]–[11], cell polarization and tissue architecture are disrupted [10] and anoikis/apoptosis is inhibited [12]–[15]. All of the above effects can contribute to tumorigenicity and, in fact, ectopic expression in rat L6 myoblasts [16] or over-expression in human Caco2 colonocytes [10] of CEA and/or CEACAM6 prior to differentiation leads to a marked decrease in the latent period for tumor formation in nude mice.

The molecular mechanism for the observed tumorigenic effects of CEA and CEACAM6 could provide insight in considering this question. The structural requirements for CEA's differentiation-blocking ability have been shown to be self-associating external domains linked to a CEA-specific GPI anchor [17], the former to effect clustering and the latter to provide activation specificity following clustering [18], [19]. CEA occupies membrane microdomains (membrane rafts), which tend to cluster as the CEA cell surface density increases as it does in many cancers (see above). CEA external domain mutants deficient in self-binding have no effect on differentiation but become effective immediately after antibody-mediated cross-linking [18]. Thus, within 5 minutes after cross-linking, integrin α5β1, a cell surface heterodimeric receptor affecting cell-extracellular matrix/cell-cell interactions involved in cell proliferation, differentiation and survival [20], becomes activated and co-localizes with CEA in larger membrane structures; rapid localization in low density membrane microdomains of ILK, AKT and MAPK and phosphorylation of AKT and MAPK are also observed [21], presumably because all of these elements occupy the same membrane rafts as CEA. Similarly, antibody-mediated cross-linking of CEACAM6 activates integrin αvβ3 in a pancreatic cancer cell line [22]. Integrin activation and subsequent activation of the PI3K/AKT and MAPK pathways have been reported by other groups to have the same effects observed here on differentiation and anoikis [23]–[25]. These results are thus consistent with the instrumental model of CEA and CEACAM6 in tumorigenesis.

The above results utilized various *in vitro* model systems involving CEA/CEACAM6 transfectants and could therefore have given findings that do not apply *in vivo*. To address this important caveat and to examine effects on tissue architecture in a valid setting, it was decided to construct transgenic mice containing human genes for CEA and CEACAM6. Mice do not possess GPI-anchored CEA family members, having only analogs of human TM-anchored CEACAM1 [26], making this experiment appropriate. Previous transgenics harboring the human CEA gene only, while showing the same pattern of tissue specific expression as in humans [27], [28], did not show a predilection for developing more tumors even when crossed with tumor susceptible mutant mice, such as APC^Min/+^ mice [29]. These mice, however, lacked CEACAM6 that is usually co-overexpressed with CEA in cancers, had transgene expression levels that could have been below those observed in human tumors and lacked upstream and downstream genomic sequences in their transgenes that could have been necessary for derangement of their expression, e.g., in colonocytes with division potential.

The above deficiencies were dealt with by creating transgenic mice containing a 187 kb insert from a bacterial artificial chromosome (BAC) that included the complete human genes for CEA, CEACAM6, CEACAM7 and CEACAM3 plus about 20–30 kb of both upstream and downstream sequences [30]. The expression patterns of these genes were almost identical to human patterns, showing normal expression of CEA and CEACAM6 in colorectal crypts with, unlike the CEA-only transgenics by Eades-Perner *et al.*, the correct gradient, i.e., higher towards the upper ends of the crypts. They also showed very low colorectal cryptal expression of CEACAM7 and no colorectal expression of CEACAM3 [30]. Two independent transgenic founders were obtained with 2 and 10 head-to-tail copies of the transgene and colonic expression levels directly proportional to their copy number [30]. Higher copy transgenics were then derived by mating the 10 copy founders to give 20 copy homozygotes and a further 2-fold increase in colonic expression. The latter expression levels were in the range observed in human colorectal carcinomas. We were thus poised to look for possible tumorigenic effects of CEA and CEACAM6 in an *in vivo* system that more closely approximated the human situation.

These transgenic mice showed dramatic expression level-dependent tumorigenic effects: with some variation in detail, the same changes in integrin α5β1, ILK and AKT as seen in the *in vitro* model systems were observed in purified colonocytes; furthermore, the mice showed inhibition of colonocyte differentiation (at least for the goblet cell lineage) and anoikis and disruption of tissue architecture characteristic of extreme hyperplasia and dysplasia.

## Results

### Construction of CEABAC Transgenic Mice

**Figure 1:**
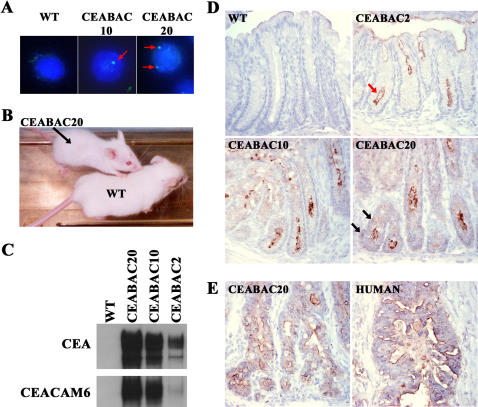
Expression of CEA and CEACAM6 in the CEABAC mouse colon. A) Images of fluorescence *in situ* hybridization (FITC-labeled CEA cDNA probes and DAPI-stained nuclei) show one nuclear spot (red arrow) for CEABAC10 and two for CEABAC20. Magnification: 1000×. B) Significant reduction visually of body size of CEABAC20 mice at 3 weeks of age. C) Immunoblots of colon protein extracts show a correlation between expression levels of CEA (detected with A20 mAb) and CEACAM6 (detected with 9A6 mAb) and CEABAC transgene copy numbers in the CEABAC mice (CEABAC2, CEABAC10 and CEABAC20). D) Immunohistochemical staining (brown staining) for human CEACAM (detected with RbαCEA) in 3 week-old mouse colons shows increasing expression levels of CEA/CEACAM6 correlated with the transgene copy number. The high expression level of CEA/CEACAM6 in the CEABAC20 mice was no longer restricted to the apical surface (red arrow points to apical surface staining; black arrows point to basolateral staining) and significant intracellular localization of CEA/CEACAM6 was also evident. E) Immunohistochemical staining (brown staining) for human CEACAM (detected with RbαCEA) in 3 month-old CEABAC20 mouse colons shows levels and patterns of CEA/CEACAM6 expression which are similar to those of human colorectal carcinomas at a similar stage of progression. WT denotes wild-type littermate.

CEABAC2 and CEABAC10 transgenic mice are independent founders containing 2 and 10 head-to-tail copies, respectively, of the 187 kb genomic DNA insert of a BAC which includes the genes for human CEA, CEACAM6, CEACAM7 and CEACAM3 [30]. The tissue specific expression patterns for these genes have been documented previously [30] and are almost identical to those in humans [1]. CEABAC20 mice with 20 copies were obtained by mating CEABAC10 mice, which are heterozygous for the transgene. *In situ* hybridization with a fluorescein isothiocyanate (FITC)-labeled CEA cDNA probe of cell nuclei showed a single spot for CEABAC10 and two spots for CEABAC20 (Figure 1A), which is consistent with the previous molecular analysis of CEABAC10 indicating head-to-tail linkage of all copies into one complex [30]. The CEABAC20 mice could be immediately recognized in litters because of their significantly smaller size (Figure 1B), which could be attributed to impaired gastrointestinal function (see below).

Immunoblot analysis for CEA and CEACAM6 expression in the colons of 3 month old mice is shown in Figure 1C. As expected, wild-type (WT) mice show no expression of these human CEACAM family members, whereas CEABAC2, CEABAC10 and CEABAC20 mice show increasing expression levels in direct proportion to their transgene copy number.

The spatial expression pattern, both qualitatively and quantitatively, was important to assess, if any of these mice were to serve as models for human colorectal carcinogenesis. Appropriately elevated expression levels in colonocytes in the proliferative zone of colonic crypts would be required for this. As an approximate estimate, immunohistochemistry for CEA/CEACAM6 was performed on colonic sections from 3 week and 3 month old transgenic mice and compared with that of human colorectal carcinomas at a common stage of progression. In concordance with the immunoblot analysis, CEABAC2, CEABAC10 and CEABAC20 mice showed increasing expression levels of CEA/CEACAM6 in the transgenic colons at 3 weeks of age (Figure 1D). However, the high expression level of CEA/CEACAM6 in the CEABAC20 mice was no longer restricted to the apical surface. Moreover, intracellular localization of CEA/CEACAM6 was more evident in the CEABAC20 mice (Figure 1D). By 3 months of age, the expression level and pattern of CEA/CEACAM6 in the CEABAC20 mice were similar to those of human colorectal carcinomas (Figure 1E).

### Molecular Changes in CEABAC Colonocytes

**Figure 2:**
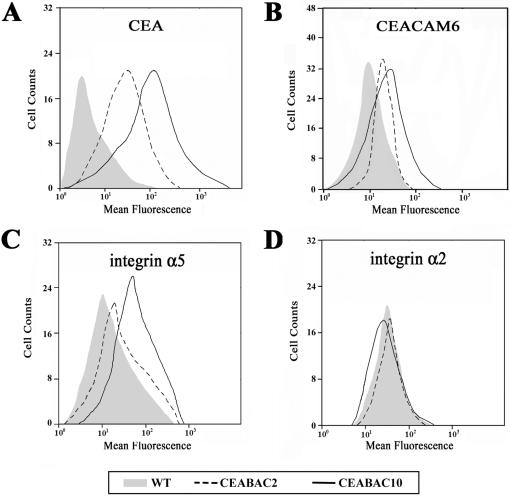
Cell surface expression of CEACAM and integrins on purified colonocytes. FACS profiles of WT (shaded area), CEABAC2 (dotted line) and CEABAC10 (solid line) purified colonocytes. A–B) FACS profiles of CEA detected by T84.66 mAb (A) and CEACAM6 detected by 9A6 mAb (B), show a higher cell surface expression of CEA and CEACAM6 in the CEABAC10 than CEABAC2 colonocytes, whereas wild-type (WT) colonocytes show only a background flurorescence. C) FACS profiles of integrin α5 detected by HMα5-1 mAb show increasing cell surface expression levels of integrin α5 with increasing CEA/CEACAM6 cell surface expression levels (A–B). D) FACS profiles of integrin α2 detected by HMalpha2 mAb show the same cell surface or even less expression levels of integrins α2.

A case for a positive change in the activation state of integrin α5β1 as a result of CEA clustering was made from previous *in vitro* studies (see Introduction). Here, using purified colonocytes from the transgenic mice, it was not technically possible to measure their binding to fibronectin, the major ligand of integrin α5β1, as an indication of its activation state. However, the cell surface level of integrin α5 was higher in CEABAC2 than in WT colonocytes and even higher in CEABAC10 (Figure 2C). This finding correlated well with the cell surface levels of CEA and CEACAM6 in the CEABAC2 and CEABAC10 mice (Figures 2A and 2B). CEABAC20 colonocytes were not included in this part of the characterization because the extensive distortion of the crypt architecture (see below) rendered their isolation too difficult using the current method. The expression level of integrin α2 showed no such increase with CEA/CEACAM6 expression (Figure 2D) and integrin αv could not be detected (data not shown). These results suggest that integrin α5β1 signaling could be increased in colonocytes from CEABAC transgenic mice and in a CEA/CEACAM6 gene dose-dependent fashion.

**Figure 3:**
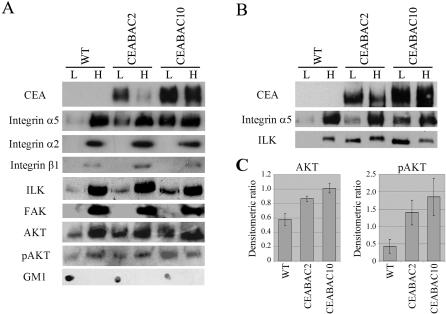
Subcellular localization of CEACAM and integrin signaling elements in purified colonocytes. A) Immunoblots of different membrane fractions of purified colonocytes derived from sucrose density gradients show a shift in subcellular localization from high density (H) towards low density (L) membrane complexes for integrin α5, ILK, total AKT and Ser473-phosphorylated AKT (pAKT) in purified colonocytes from CEABAC2 and CEABAC10 mice relative to WT littermate mice. No such shift was observed for integrin α2, integrin β1 and FAK. GM1 is a marker for membrane raft-containing membrane fractions. Note that these immunoblots are representative of three independent experiments using at least 6 mice per mouse group. B) Antibody-mediated cross-linking of CEA and CEACAM6 with J22 mAb in the purified colonocytes accentuates the shift of ILK to the less dense membrane fractions shown in A. C) Densitometric analysis of immunoblots for AKT and pAKT shown in A. The mean densitometric ratios (the intensity of band in L divided by the one in H) from three independent experiments show a statistical significant shift of AKT and pAKT to the low density membrane complexes in the CEABAC colonocytes (*P*<0.001 for AKT and *P*<0.005 for pAKT). Error bars  =  SEM (N = 3).

In agreement with the latter supposition, a shift in subcellular localization towards less dense membrane complexes typical of membrane rafts for integrin α5, ILK and AKT and an increase in phosphorylation for AKT were observed in purified colonocytes from CEABAC2 and CEABAC10 mice relative to WT littermate mice (Figure 3A & C), as was previously seen for these elements in various *in vitro* systems [21]. No such changes were observed for the integrin α2 subunit, the integrin β1 subunit or FAK (Figure 3A). However, since the overall detection level of integrin β1 (in contrast to integrin α2 and FAK) was very low and no visible signal was detected in the low-density membrane fractions, the failure to see a shift in the subcellular localization of integrin β1 was questionable. In addition, the β1 subunit is shared by many α subunits and so might not be expected to show large changes. Similarly, PI3K was undetectable in this experiment (data not shown).

These increases were understandably less dramatic than those observed *in vitro* in which the events were synchronized by cross-linking cell surface mutant CEA molecules that lacked self-adhesive external domains [21]. Here, antibody-mediated cross-linking of CEA and CEACAM6 in the purified colonocytes accentuated the shift of ILK towards less dense membrane complexes (Figure 3B), which is consistent with the suggestion that they arose as a consequence of CEA/CEACAM6 clustering leading to integrin α5β1 activation. Regardless of the verity of the latter interpretation, the results provide evidence for the activation of AKT, an acknowledged consequence of integrin activation [20], [25].

### Cellular Changes in CEABAC Colonocytes

**Figure 4:**
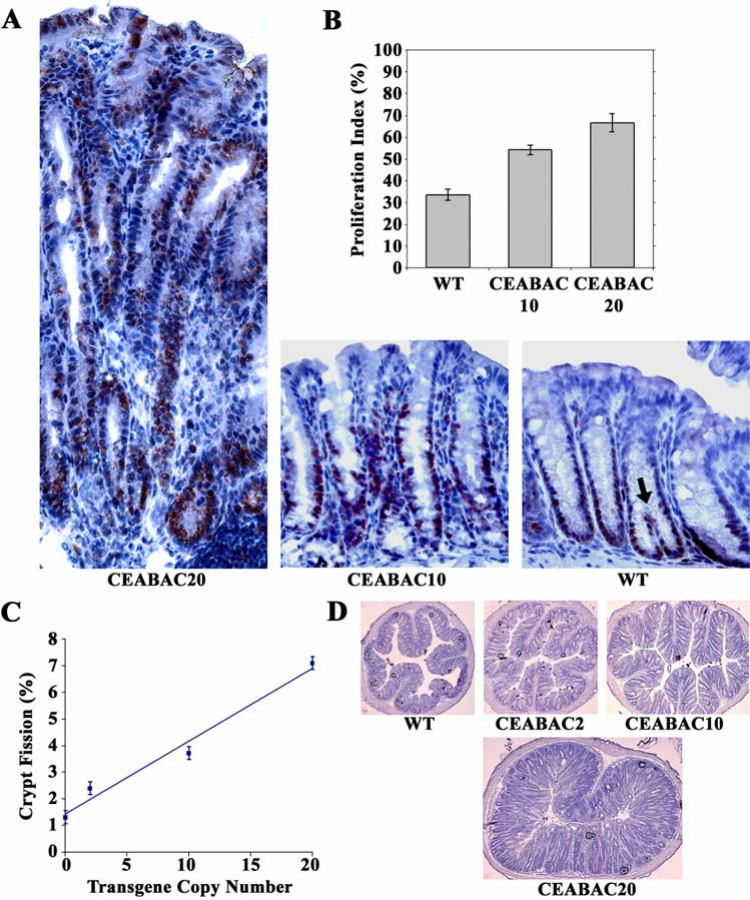
Hyperproliferation in CEABAC mouse colonic epithelium at 3 weeks of age. A) Extension of the proliferative zones in colonic crypts is shown for both CEABAC10 and CEABAC20 by PCNA staining. Lengthening of crypts, poor cryptal alignment, increase of peri-cryptal stroma, and elongation of nuclei are noted in CEABAC20 mice, indicating hyperplastic changes. Arrow shows an example of crypt fission used to calculate percent crypt fission for C. B) Proliferation indices estimated by PCNA staining in A show significant increases for both CEABAC10 and CEABAC20 from WT mice (*P*<0.0001). C) Percent crypt fission plotted against transgene copy-number shows a linear curve with a correlation coefficient of 0.977; each data point is significantly different from the others (*P*<0.0001). D) Cross sections of colons show increasing epithelial content with increasing CEABAC transgene copy number (i.e. CEA/CEACAM6 expression levels). Magnification: 400× (A), 100× (D). Staining: hematoxylin. Error bars represent standard errors of the mean (N = 9). WT denotes wild-type littermate.

**Figure 5:**
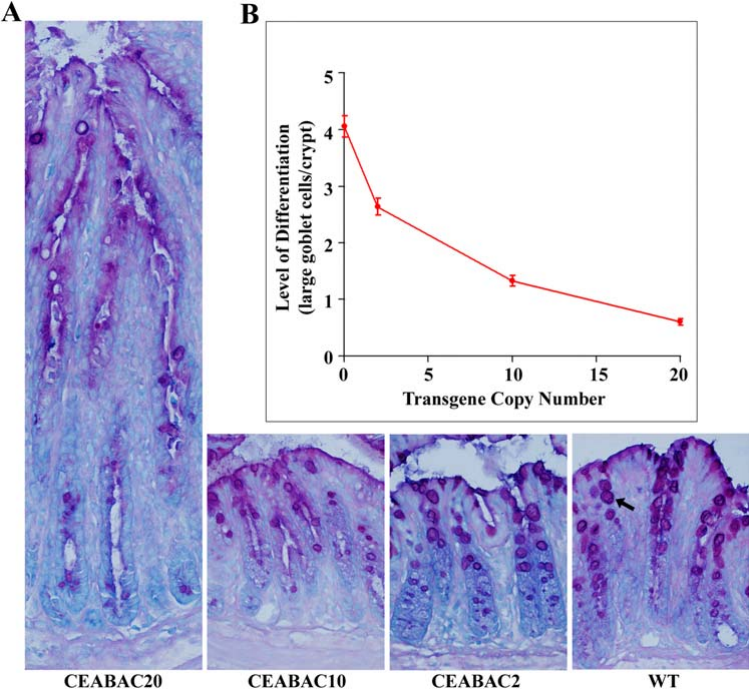
Differentiation block in CEABAC mouse colonic epithelium at 3 weeks of age. A) Mucin staining of colonic crypts of WT, CEABAC2, CEABAC10, and CEABAC20 mice shows a progressively decreasing number of visible purplish-colored differentiated goblet cells (indicated by an arrow in the WT colonic crypt) correlated with increasing CEABAC transgene copy number. Lengthening of crypts, poor cryptal alignment, increase of peri-cryptal stroma, absence of bluish cytoplasm, abundance of small purplish mucinous vacuoles and elongation of nuclei are noted in CEABAC20 mice, indicating hyperplastic changes. Magnification: 400×. Staining: AB/PAS/MG. B) Number of normal-size goblet cells per crypt plotted against transgene copy-number; each data point is significantly different from the others (*P*<0.0001). 35%, 67% and 85% (95% after considering the 3-fold increase in crypt length) reductions relative to WT mice (i.e. 0 transgene copy) are obtained for CEABAC2, CEABAC10 and CEABAC20 mice, respectively. Error bars represent standard errors of the mean (N = 9). WT denotes wild-type littermate.

What are the cellular consequences *in vivo* of the increase in cell surface density of integrin α5 and the consequent downstream molecular activation events that arise from the presence of the CEABAC transgenes in colonocytes? A transgene copy number-dependent increase in the colonocyte proliferation index indicated by PCNA staining was observed in colonic crypts from 3 week-old CEABAC transgenics relative to WT littermates (Figure 4B). This was due to the progressive extension (with CEABAC dose) of the proliferative zone towards the upper parts of the crypts reaching the full length of the crypts in CEABAC20 mice (Figure 4A). This effect resulted in an overall increase in the length of the CEABAC20 crypts (Figures 4A and 5A). There was also a marked CEABAC dose-dependent increase in the incidence of crypt fission (Figure 4C), the process whereby new crypts are created in the colon [31].

Lastly, a strong CEABAC dose-dependent decrease in the differentiation of colonocytes, at least for the goblet cell lineage in the crypts, was observed in the transgenic mice (Figure 5), consistent with the pan-inhibition of cellular differentiation noted in various *in vitro* systems [9]–[11].

### Hyperplastic and Dysplastic Changes in CEABAC Colons

**Figure 6:**
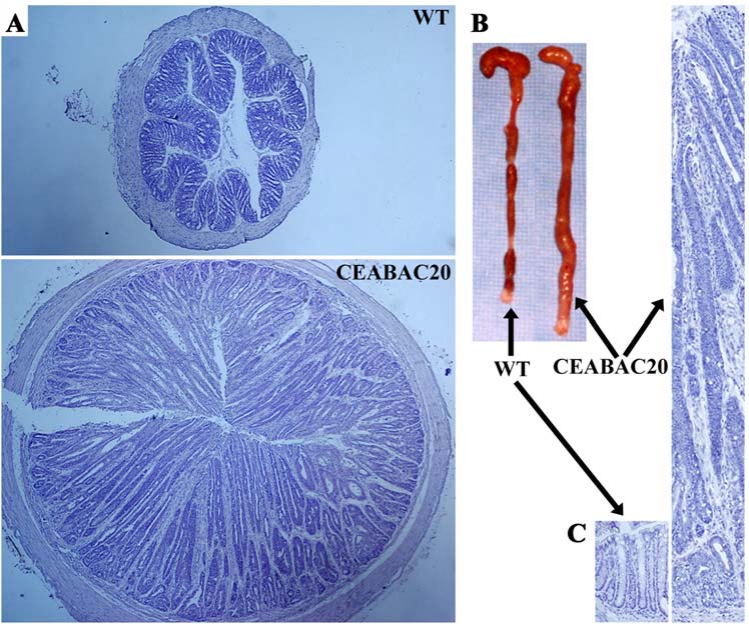
Massive colonic tumors in 3 month-old CEABAC20 mice. A) Cross sections of colon; note the much larger size of the CEABAC20 vs WT colons. B) Dramatic increase of colon mass and absence of fecal pellets in CEABAC20 over the entire colon. C) Colonic crypts; note the dramatic lengthening of intensely stained crypts in CEABAC20. Magnification: 40× (A), 100× (C). Staining: hematoxylin (A, C).

A progressive shift with CEABAC dose towards a deranged tissue architecture featuring more mucosal tissue and less luminal space was seen in cross sections of colons from 3 week-old transgenic mice (Figure 4D). This effect became quite dramatic in CEABAC20 mice by 3 months of age, where 100% of the mice showed grossly enlarged colons with crypts up to 10 times normal length and with virtually no central lumen (Figure 6). These mice were considerably smaller than normal (Figure 1B), had persistent diarrhea and rectal bleeding, and died prematurely from the first weeks of life to about 9 months maximum; all animals died with rectal prolapse. The effects were more pronounced in female, especially pregnant, mice than in males.

**Figure 7:**
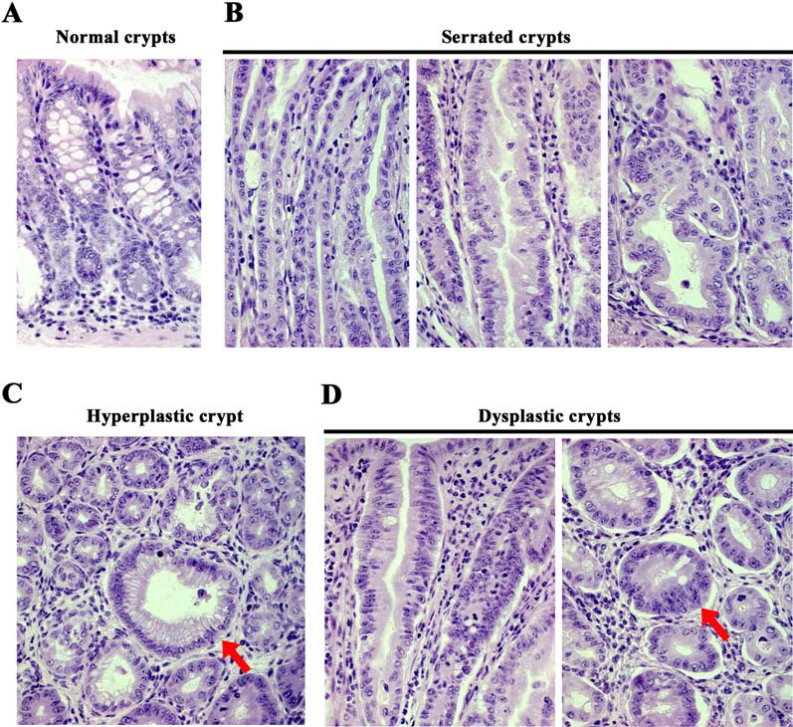
Histopathological findings in CEABAC20 tumors including serrated adenomatous and dysplastic morphology. A) Longitudinal section of normal colonic crypts. B) Longitudinal sections of serrated colonic crypts showing upper third of the elongated crypts (left panel), sawtooth like in-folding at the mid-crypt (middle panel) and marked branching at the crypt base (right panel). C) Cross-section of a hyperplastic crypt (red arrow) showing numerous small mucinous vacuoles. D) Longitudinal section (left panel) and cross-section (right panel) of dysplastic crypts showing elongated nuclei and nuclear stratification (red arrow). There is also evidence of myofibroblastic (spindle cell) proliferation in the lamina propria in C and D. Magnification: 400×. Staining: hematoxylin and eosin.

Histological analysis of colonic mucosa from the 3 month-old CEABAC20 mice showed a non-focal continuous mosaic of severe hyperplasia, crypt serration and dysplasia (Figure 7). Dysplastic features included colonocyte stratification, displaced nuclei with prominent nucleoli and loss of polarization.

**Figure 8:**
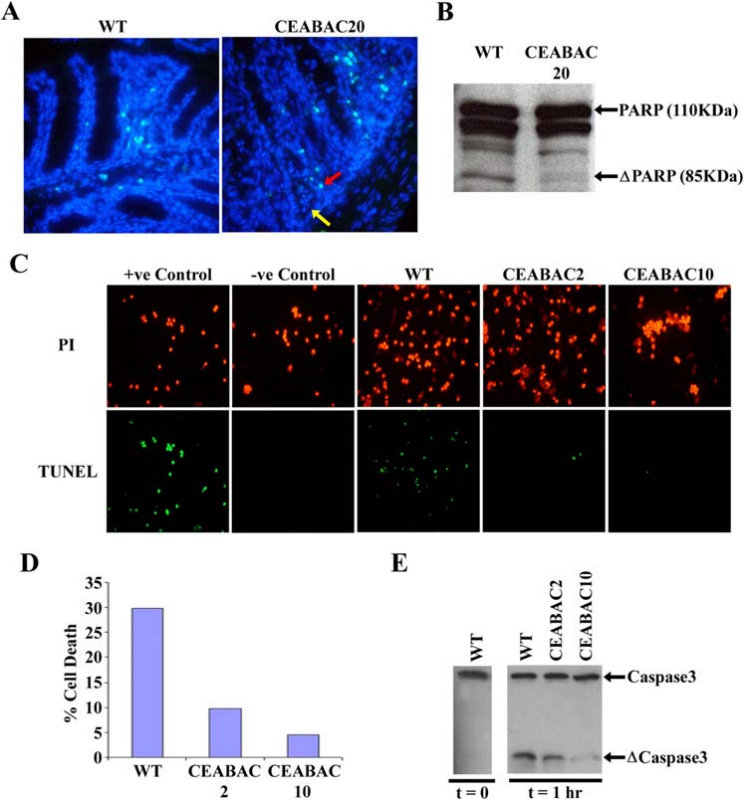
Inhibition of apoptosis/anoikis in CEABAC mice. A) TUNEL assays on formaldehyde-fixed frozen sections of WT and CEABAC20 colons. Cyan (red arrow): apoptotic nuclei; Blue (yellow arrow): non-apoptotic nuclei. Note the presence of non-apoptotic anchorless cells, which are CEA/CEACAM6-positive (data not shown), in the crypt lumens of CEABAC20 colons (yellow arrow). Magnification: 400×. Staining: DAPI. B) Immunoblots of colon protein extracts for PARP show reduced cleavage product of PARP (ΔPARP) indicating an overall reduction of apoptosis in CEABAC20 mouse colons. C) TUNEL assays on purified colonocytes show a marked inhibition of anoikis of CEABAC colonocytes compared to WT colonocytes after 3 hours in single cell suspension. Negative control (freshly purified WT colonocytes) is completely TUNEL negative. Positive control (freshly purified WT colonocytes treated with DNAseI) is TUNEL positive. D) Apoptotic indices estimated from 3 independent experiments shown in C. E) Immunoblots of colonocyte lysates from anoikis experiment for caspase-3 show a baseline cleavage of caspase-3 (ΔCaspase3) at time 0 (freshly purified colonocytes) and a marked reduction of caspase-3 cleavage at time 1 hr (after 1 hour in single cell suspensions) for CEABAC mice, indicating inhibition of anoikis in the CEABAC colonocytes.

A further change, characteristic of neoplasia, was the inhibition of anoikis, the apoptotic process that normally removes cells that are not properly anchored to the extra-cellular membrane and which therefore acts as a quality control mechanism for tissue architecture [24]. If the CEA/CEACAM6-mediated disruption of tissue architecture is to persist, this process needs to be inhibited. In fact, crypts from 3 month-old CEABAC20 mice often showed CEA/CEACAM6-positive anchorless cells in their lumens that were negative for staining by the TUNEL assay; such cells were not seen in WT crypts (Figure 8A). Moreover, protein extracts from CEABAC20 colonic mucosa showed less PARP cleavage than from WT mucosa (Figure 8B), suggesting that the proportion of cells undergoing apoptosis was less in the CEABAC20 mice than in WT mice. In addition, colonocytes purified from isolated crypts of even the reduced CEABAC dose CEABAC2 and CEABAC10 mice, when maintained in suspension, showed a dramatic reduction in apoptosis (Figures 8C and 8D). At the molecular level, the cleavage of caspase-3, which represents a hallmark of apoptosis [32], was also reduced in the suspended CEABAC2 and CEABAC10 colonocytes (Figure 8E).

## Discussion

The fact that CEA and/or CEACAM6 are over-expressed in so many human cancers has led us and others to suggest that this effect could contribute to tumorigenesis [4], [10], [12], [33]. However, the normally elevated expression of these molecules in differentiated epithelial cells presents a conundrum. Although evidence for tumorigenic effects of CEA and CEACAM6 has been documented in many diverse systems *in vitro* (see Introduction), the latter could be criticized for being model systems that fail to represent the real situation *in vivo*. This study, however, utilizing transgenic mice expressing human CEA and CEACAM6 genes with the human tissue specific pattern but with increasing expression levels to the point of those found in human carcinomas, represents perhaps the closest approximation to the human situation yet investigated. The results were striking in that purified colonocytes from these mice showed CEA/CEACAM6 expression level-dependent molecular changes involving integrin activation similar to those documented in the *in vitro* systems; tumorigenic cellular changes were observed such as increased proliferation and inhibition of differentiation and anoikis/apoptosis; and, finally, these cellular changes were mirrored by dramatic changes in colonic tissue architecture featuring severe hyperplasia and dysplasia, to the point of rendering the entire colon a massive tumor-like structure. The resolution of the above conundrum is suggested to be that elevated expression of CEA/CEACAM6 in colonocytes *prior* to their differentiation and loss of division potential is required for the manifestation of their tumorigenic effects.

### Molecular Basis of Tumorigenic Changes

We suggest that the initial molecular events involved in these effects consist of CEA/CEACAM6-mediated clustering of membrane microdomains that specifically contain these adhesion molecules, integrin α5β1, and a limited repertoire of cytoplasmic signaling elements, including ILK, PI3K and AKT. The co-clustering causes integrin α5β1 to become activated, along with the PI3K/AKT pathway, by successive waves of inside-out and outside-in signaling [34], [35] resulting in inhibition of cell differentiation and anoikis and stimulation of cell proliferation–the latter previously documented by other groups to be caused by integrin activation [36], [37]; ultimately, tissue architecture is profoundly perturbed. Much of the evidence for this suggested model comes from detailed studies of the more experimentally amenable *in vitro* systems [19], [21], [22], [38] and the fact that these are mirrored by key changes in purified colonocytes from the CEABAC transgenics implies that it could apply in this situation too.

The question arises as to whether these effects could have been due to a random insertional mutation of an endogenous gene rather than to direct effects of the elevated expression levels of CEA and CEACAM6. The former is unlikely, however, for the following reasons. First, random mutation of endogenous genes by transgenesis is an uncommon event [39]. Secondly, the probability of mutation of a gene resulting in an anticipated effect, i.e., specifically colorectal tumorigenesis, is even lower. Thirdly, to our knowledge, such dramatic tumorigenic effects have never been reported in any knockout models. Lastly and most importantly, both independently produced transgenic lines (CEABAC2 and CEABAC10), which would have different sites of integration, showed similar tumorigenic changes directly related quantitatively to expression level.

It is not clear from this work, however, which of the CEACAMs present in the CEABAC are responsible for these effects. Since, as in humans, CEACAM7 is expressed only at very low levels in these transgenics' colons [30] and is under-expressed rather than over-expressed in human colorectal cancers [1], and CEACAM3 is not at all expressed in the colon [1], [30], it seems very likely that CEA and/or CEACAM6 are responsible and, in fact, both of the latter genes have been shown to elicit tumorigenic effects in various model systems *in vitro* and in xenograft mouse models [9]–[15], [40]. It is possible that over-expression of *both* CEA and CEACAM6 are required for these effects *in vivo*, which would explain the failure to see them in CEA-only transgenics [27]–[29], although it is equally possible that the CEA colonocyte expression levels in the latter were too low or that the CEACAM6 expression is the key determinant.

With the likely assumption that the effects are due to CEA and CEACAM6, clustering of these molecules as their concentration increases by self-binding of their adhesive external domains [18] is suggested to be the initial event leading to the changes in cellular state observed here. This suggestion is supported by previous work with several cell lines expressing a CEA mutant that is defective in self-binding. None of the molecular events, including integrin α5β1 activation, or the cellular events, including inhibition of differentiation were produced by this mutant, but all these events were promptly restored by cross-linking with specific antibodies [18], [21]. In this study, the tumorigenic effects were strongly gene dose dependent, which would be expected if they depended on self-binding and clustering, which would be a positive power function (at least a square function) of cell surface molecular concentration. This was most apparent in the case of the disruption of colonic tissue architecture, which was extreme in the case of the CEABAC20 mice. It is of interest that the CEABAC2 and CEABAC10 mice, which showed more moderate colonic changes, were nevertheless much more prone to produce carcinogen-induced focal colorectal tumors than WT mice [41].

Evidence for the involvement of integrin α5β1 activation, previously shown in *in vitro* systems, was obtained in the observation of a CEABAC dose-dependent increase in the colonocyte surface concentration of this integrin relative to WT littermates and integrin α2 surface concentration. We speculate that this increase is due to an initial activation of integrin α5β1, as observed *in vitro*, followed by an increase in cell surface level, a positive feedback control loop previously documented for integrins in many other systems [42], [43]. If so, it could imply that the colonocyte integrin α5β1 molecules are not only increased in number but also in activation state, although this would obviously require direct confirmation. The CEABAC mouse colonocytes showed evidence for the activation of AKT but, in preliminary experiments, no evidence for activation of the Wnt-signaling pathway could be demonstrated, in that no nuclear accumulation of β-catenin was seen (Chan and Stanners, unpublished data).

### Relevance to Human Colon Carcinogenesis

The most striking and unexpected feature of the present results is first, the magnitude of the effects, which are arguably greater than those seen for any single oncogene or inactivated tumor suppressor gene expressed in animals and second, the fact that this tumorigenic phenotype involves the entire colon in a diffuse mosaic pattern of changes (Figures 6 and 7) in 100% of the CEABAC20 mice. CEA, and possibly CEACAM6, over-expression has been shown to be mutagenic [16], [41], leading to the rapid outgrowth of rat L6 tumors with a dramatically reduced latent period, reduced even more than that observed with the expression of a combination of v-myc and Bcl2 [16]. If random mutations were required in the CEABAC20 mice, however, the neoplastic changes observed would be focal, as is the case for all other published mouse models with tumorigenic genetic alterations. The present case may bear some resemblance to that of mucin Muc2 deletion in mice [44], in which relatively small non-focal increases in proliferation and decreases in apoptosis were observed in the entire gastrointestinal tract; in this case, focal carcinomas appeared later in life, presumably due to secondary mutations. This is actually the case for the CEABAC2 and CEABAC10 mice which showed a marked increase in focal carcinomas after azoxymethane treatment [41]. In the CEABAC20 mice, on the other hand, the expression level of CEA and CEACAM6 is high enough to drive the abnormal growth of colonic crypts demonstrated by the extreme cellular changes within a given crypt. This abnormal and uncontrolled growth pattern leads to a complete disruption of normal tissue architecture and a large increase in crypt length. In fact these primary changes are so dramatic in the CEABAC20 mice that any focal carcinomas would likely have little selective growth advantage and, in any case, the animals tend to die from digestive complications before about 6 months of age, thus not allowing a long term assessment. Since human colonic tumorigenesis is in fact focal, we suggest that, unlike the situation here where every cell has multiple copies of CEA and CEACAM6 genes, their over-expression itself in the adult human colon could occur by focal up-regulation in expression as observed in the carcinogen-induced colon tumors in CEABAC2 and CEABAC10 mice [41]. We therefore propose that the increase in proliferation and inhibition of differentiation and anoikis together with the disruption of tissue architecture, induced by CEA/CEACAM6 over-expression in colonocytes, puts them in a state that is more susceptible to effects of other oncogenic lesions, thus representing a significant contribution to malignant transformation [16]. Note that this model does not require structural mutations in CEA or CEACAM6 themselves and, in fact, such mutations are generally not observed in cDNAs derived from human tumors over-expressing these molecules [1].

Another interesting feature of these tumors was their resemblance to mixed hyperplastic polyps, serrated polyps and serrated adenomas in human patients, which are subtypes of pre-cancerous lesions [45], [46] that show increased expression of CEA [47], [48]. In contrast to conventional adenomas in which perturbation of the Wnt-signaling pathway is commonly believed as the initiating event [49], [50], this serration is a consequence of alteration in cell proliferation and apoptosis leading to inactivation of DNA repair genes by promoter methylation and secondary mutations that cause tumor progression [46]. Based on the proliferative and anti-apoptotic effects of CEA/CEACAM6 shown in the CEABAC mice, it is thus possible that this could represent the first animal model for this type of human colorectal neoplasia.

However, the non-focal concomitant dysplasia observed in the CEABAC20 mice requires comment. The traditional view is that hyperplasia is a *dead-end* state that does not progress to dysplasia. Although the serrated pathway allows such a transition, only a small proportion of hyperplastic lesions transform to dysplastic serrated adenomas after acquiring other gene mutations, such as hMLH1 or MGMT [46]. The rather frequent non-focal transition to colonic tissue with features of dysplasia observed in CEABAC20 mice suggests that over-expression of CEA and/or CEACAM6 to a critical threshold level alone is sufficient to initiate the transformation of a normal epithelium to a state of dysplasia. It thus appears that homotypic adhesion molecules with their self-binding external domains can effect this by clustering without the need for mutations, either in themselves or in other elements. Secondary mutations [whose incidence is stimulated by this state [16]], however, would be required to ensure rigorous heritability.

### Significance for Human Cancer

If these results can be extrapolated to human colorectal cancer, in which up-regulation of CEA and CEACAM6 occurs as an early event in adult colonic epithelium [4], [8], [51], the magnitude of the tumorigenic effects observed here would imply a very significant contribution to the malignant phenotype. Thus, clinically, cancer treatments should aim to reduce their levels and should not further up-regulate them to enhance tumor targeting. Perhaps more importantly, strategies designed to reverse their biological effects rather than to use them as inert targets should be considered. In fact, reduction of their expression has been shown to reverse some tumor phenotypes [13], [14], [52] and to restore sensitivity to chemotherapeutic agents [53], as have *declustering* agents [18]. Thus, development of agents that inhibit expression and function of these proteins could prove beneficial to clinical practice.

## Materials and Methods

### Animals

CEABAC transgenic mice (CEABAC2 and CEABAC10 lines, bearing 2 and 10 copies of the CEABAC transgene, respectively) were produced and maintained on the FVB strain background. Genotypes of mice were determined by PCR as described elsewhere [30]. CEABAC20 mice (bearing 20 copies of the CEABAC transgene) were produced from mating two CEABAC10 mice. Homozygous transgenic mice were identified by fluorescence *in situ* hybridization of the CEABAC in splenocytes (see below).

### Antibodies

Rabbit polyclonal antibodies (Ab) used were: anti-CEA Ab (RbαCEA) which recognizes all human CEACAM family members; anti-integrin α5 Ab (H-104), anti-integrin β1 Ab (M-106) and anti-PARP Ab (H-250) which detects the C-terminus of PARP-1 and PARP-2 (Santa Cruz Biotechnology Inc., Santa Cruz, CA); anti-FAK Ab (Upstate Cell Signaling, Charlottesville, VA); anti-Akt Ab which detects the total level of Akt1, Akt2 and Akt3, anti-Phospho-Akt (Ser473) Ab which detects Akt1 only when phosphorylated at the Ser473 residue and also Akt2 and Akt3 when phosphorylated at the corresponding residues, anti-caspase-3 Ab which detects full length caspase-3 and the large fragment of cleaved caspase-3 (Cell Signaling Technology Inc., Beverley, MA). Mouse monoclonal antibodies (mAb) used were: anti-CEA mAb (J22 and A20) which recognize the internal domains of CEA and CEACAM6 and the N-terminal domain of CEA, respectively [54]; anti-CEA mAb (T84.66) which recognizes only CEA (ATCC, Manassas, VA); anti-CEACAM6 mAb (9A6) which recognizes only CEACAM6 [4]; anti-ILK mAb (BD Biosciences, Mississauga, Canada); and anti-PCNA mAb (PC10) (DAKO Diagnostics Canada Inc., Mississauga, Canada). Hamster monoclonal antibodies used are: anti-integrin α5 mAb (HMα5-1) (BD Biosciences) and anti-integrin α2 mAb (HMalpha2) (Chemicon International Inc., Temecula, CA).

### Fluorescence *In Situ* Hybridization and TUNEL Assay

For fluorescence *in situ* hybridization (FISH), freshly isolated splenocytes were treated as previously described [55]; DIG-labeled CEA cDNA probes were generated using the High Prime DNA Labeling Kit (Roche Diagnostics Inc., Laval, Canada), and detection was performed using the Fluorescent Antibody Enhancer Set for DIG Detection (Roche Diagnostics Inc.). The terminal deoxynucleotidyl transferase (TdT)-mediated dUTP Nick-End-Labeling (TUNEL) assay was used to detect apoptotic cells according to the protocol provided by the APOAlert™ DNA Fragmentation Assay Kit (BD Biosciences). Fluorescent cells were visualized using a fluorescent light microscope.

### Isolation of Mouse Colonic Epithelial Cells

Colonic epithelial cells were isolated from mice using a protocol modified from one previously described for human samples [56]. Briefly, 6 intact mouse colons were flipped inside-out and incubated for 30 minutes in a 1.3 mM DTT/PBS solution at room temperature and then 10 minutes in a 1.5 mM EDTA/PBS solution at 37°C. Intact colonic crypts were separated from the underlying layers by vigorous shaking, collected using a 40-µm nylon cell strainer (BD Biosciences) and treated with dispase (1.2 mg/ml) for 10 minutes to give a single-cell suspension.

### Detection of cell surface levels of CEA, CEACAM6 and integrins

Cell surface levels of CEA, CEACAM6 and integrins on purified colonic epithelial cells were determined by fluorescence-activated cell sorting (FACS) analysis, as described elsewhere [41], using T84.66, 9A6, HMα5-1, and HMalpha2 Abs.

### Anoikis Assay for Purified Colonic Epithelial Cells

Single cell suspensions of purified colonocytes were incubated in suspension in serum-free DMEM medium for 1 or 3 hours at 37°C. The extent of apoptosis was assessed by immunoblot analysis of caspase-3 cleavage after 1 hour (see below) and by the TUNEL assay after 3 hours (see above). Baseline apoptosis was determined immediately after purification. For a positive control of the TUNEL assay, cells were pre-treated with DNAse I (1 µg/ml). For a negative control, TdT was omitted in the TUNEL reaction. Apoptotic index was expressed as a percentage of apoptotic cells over the total number of cells counted.

### Isolation of Membrane Rafts

Membrane rafts were isolated from 5–10 × 10^6^ purified colonic epithelial cells by isopycnic sucrose density gradient centrifugation; membrane raft-containing fractions were identified by the presence of GM1 using dot-blots, as described previously [21].

### Immunoblot analysis

Crude colonic tissue extracts were obtained by scraping colonic mucosa away from the underlying layers, homogenizing with PBS on ice and lysing with SDS lysis buffer (100 mM Tris pH 8, 10% glycerol, 2% SDS). Colonic epithelial extracts were obtained by lysis of purified colonic epithelial cells (see above for isolation) in RIPA lysis buffer (20 mM Tris-HCl pH 8, 140 mM NaCl, 2 mM EDTA pH 8, 10 µg/ml aprotinin, 10 µg/ml leupeptin, 100 µg/ml PMSF, 1 mM NaVO_3_, 10 mM NaF). Proteins from crude colonic tissue extracts, purified colonic epithelial extracts or sucrose gradient fractions were detected by immunoblot analyses, as described elsewhere [21].

### Histological analysis and immunohistochemistry

For cryosection, freshly collected tissues were fixed with 4% paraformaldehyde and processed as described elsewhere [30]. For paraffin-embedded sections, freshly excised tissues were fixed in Glyo-Fixx (20% ethanol, 5% glyoxal, 1% propanol and 1% methanol) for 48 hours at room temperature and processed for embedding in paraffin blocks for sectioning. For general histopathology, frozen sections were stained with hematoxylin and paraffin-embedded sections were stained with hematoxylin and eosin [57]. To assess colonocyte differentiation, frozen sections were stained by the “alcian blue-periodic acid Schiff” method to detect mucin, followed by methyl green staining to detect nuclei (AB-PAS-MG) [57]. For immunohistochemistry, frozen sections were probed with RbαCEA antibodies as described elsewhere [30]. Actively proliferating cells were detected using HRP-conjugated mouse monoclonal anti-PCNA antibody.

### Differentiation and Proliferation Analyses

For differentiation and proliferation analyses, animals were sacrificed at exactly postnatal day 21 to minimize growth variation. Percent crypt fission was scored as the percentage of forked dividing crypts over the total number of crypt bases for about 100 crypts. Proliferation index was scored as the number of PCNA-positive nuclei divided by total number of nuclei in about 10 individual crypts. The level of differentiation was scored as the number of large goblet cells (mucin vacuole of more than 10 µm) over the total number of crypt bases for about 100 crypts.
